# Methodical Challenges and a Possible Resolution in the Assessment of Receptor Reserve for Adenosine, an Agonist with Short Half-Life

**DOI:** 10.3390/molecules22050839

**Published:** 2017-05-19

**Authors:** Judit Zsuga, Tamas Erdei, Katalin Szabó, Nora Lampe, Csaba Papp, Akos Pinter, Andras Jozsef Szentmiklosi, Bela Juhasz, Zoltán Szilvássy, Rudolf Gesztelyi

**Affiliations:** 1 Department of Health Systems Management and Quality Management for Health Care, Faculty of Public Health, University of Debrecen, Nagyerdei krt. 98, H-4032 Debrecen, Hungary; zsuga.judit@med.unideb.hu (J.Z.); dr.papp.csaba@gmail.com (C.P.); 2 Department of Pharmacology and Pharmacotherapy, Faculty of Medicine, University of Debrecen, Nagyerdei krt. 98, H-4032 Debrecen, Hungary; erdei.tamas@pharm.unideb.hu (T.E.); szabo.katalin@pharm.unideb.hu (K.S.); lampenori@gmail.com (N.L.); ajszm948@gmail.com (A.J.S.); juhasz.bela@med.unideb.hu (B.J.); szilvassy.zoltan@med.unideb.hu (Z.S.); 3 Institute of Mathematics, Faculty of Science and Technology, University of Debrecen, Egyetem ter 1, H-4032 Debrecen, Hungary; apinter@science.unideb.hu

**Keywords:** adenosine, CPA, FSCPX, NBTI, A_1_ adenosine receptor, receptor reserve, receptorial responsiveness method, RRM

## Abstract

The term receptor reserve, first introduced and used in the traditional receptor theory, is an integrative measure of response-inducing ability of the interaction between an agonist and a receptor system (consisting of a receptor and its downstream signaling). The underlying phenomenon, i.e., stimulation of a submaximal fraction of receptors can apparently elicit the maximal effect (in certain cases), provides an opportunity to assess the receptor reserve. However, determining receptor reserve is challenging for agonists with short half-lives, such as adenosine. Although adenosine metabolism can be inhibited several ways (in order to prevent the rapid elimination of adenosine administered to construct concentration–effect (E/c) curves for the determination), the consequent accumulation of endogenous adenosine biases the results. To address this problem, we previously proposed a method, by means of which this bias can be mathematically corrected (utilizing a traditional receptor theory-independent approach). In the present investigation, we have offered in silico validation of this method by simulating E/c curves with the use of the operational model of agonism and then by evaluating them using our method. We have found that our method is suitable to reliably assess the receptor reserve for adenosine in our recently published experimental setting, suggesting that it may be capable for a qualitative determination of receptor reserve for rapidly eliminating agonists in general. In addition, we have disclosed a possible interference between FSCPX (8-cyclopentyl-*N^3^*-[3-(4-(fluorosulfonyl)benzoyloxy)propyl]-*N*^1^-propylxanthine), an irreversible A_1_ adenosine receptor antagonist, and NBTI (S-(2-hydroxy-5-nitrobenzyl)-6-thioinosine), a nucleoside transport inhibitor, i.e., FSCPX may blunt the effect of NBTI.

## 1. Introduction

The term receptor reserve, first introduced and used in the traditional receptor theory [1], can be considered as an integrative measure of the response-inducing ability of the interaction between an agonist and a receptor system, the latter of which consists of a receptor specific for the given agonist and a postreceptorial signaling that can be activated by the particular agonist-receptor complex. Although some recent models of agonist action do not use this term, the underlying phenomenon, i.e., the stimulation of a submaximal fraction of receptors apparently elicits the maximal effect (in certain cases), is widely known [2,3,4]. Determination of receptor reserve is based on this phenomenon, during the course of which a fraction of receptors is irreversibly inactivated in a way that the remaining fraction retains its functional integrity. The greater the receptor reserve, the greater the fraction of receptors need to be inactivated in order to achieve detectable diminution of maximal response is [4,5]. Thus, the measure of receptor reserve is the “resistance” (or “inertia”) of the receptor system, activated with a given agonist, against an intervention that reduces the number of operable receptors, in terms of the achievable maximal response.

Traditionally, the receptor reserve is thought to be determined by the agonist, tissue and effect measured [6,7]. Rephrasing this classical approach, the receptor reserve depends on three factors of the concentration–effect (E/c) relationship: agonist (exhibiting efficacy), receptor system (possessing different functional states for the receptor and a tissue-dependent signal amplification machinery), and effect (indicative of the particular signaling pathways activated).

The physiological significance of the phenomenon may be that a great receptor reserve allows an endogenous agonist to produce rapid activation of its receptor [4], or more generally, to produce fast activation of a particular receptor system. Information about receptor reserve has high utility when predicting the behavior of an agonist in a tissue. If receptor reserve in a tissue is small, only high-efficacy agonists can evoke a significant effect (acting often as a full agonist), whereas low-efficacy agonists cannot elicit an effect (or at most they behave as a partial agonist). In turn, if receptor reserve is great, even low-efficacy agonists are able to generate a significant effect (moreover, sometimes they may act as a full agonist). Thus, application of low-efficacy agonists can ensure tissue selectivity in a sense that their effect will only be significant in tissues possessing great receptor reserve. Therapeutic potential of this phenomenon has been implicated [3,8,9,10,11].

Due to the multifactorial origin of receptor reserve, it is worthwhile assessing it for every agonist, receptor system (tissue) and effect having pathophysiological importance [3,4,12]. The simplest index for receptor reserve is the pharmacological shift ratio (PSR) being equal to the quotient of agonist concentrations needed for half-maximal receptor occupancy (*K_A_*) and half-maximal effect (*EC*_50_): PSR = *K_A_*/*EC*_50_ [1]. A more sophisticated index is the percentage receptor reserve (*RR_%_*) that is the difference of effect (*E_%_*) and receptor occupancy (*ρ_%_*), both expressed as a percent of their maximums: *RR_%_* = *E_%_* − *ρ_%_* [12]. *RR_%_* addresses the essence of receptor reserve, i.e., stimulation of a given percent of total receptor population can elicit a higher percent of maximal effect. In other words, the receptor reserve refers to the percent of receptors not required for the production of maximal effect (being “spare receptors”) [4]. As shown, *RR_%_* depends on the particular value of *E_%_*, the latter of which is the asymptotic maximum of *RR_%_*. *RR_%_* is usually computed for arbitrary *E_%_* values, e.g., for the half maximal or (near) maximal effect. For the above-mentioned calculations, at first the *K_A_* values should be determined based on evaluating E/c curves (generated with a given agonist, in a given tissue and with measuring a given effect). A characteristic feature of this procedure is that these E/c curves should be constructed both in the absence and presence of an irreversible antagonist against the investigated receptor (preferably at a concentration that is able to significantly reduce the achievable maximal effect).

In an earlier study, we investigated the receptor reserve belonging to the direct negative inotropic effect (that evolves without previous β-adrenergic stimulation) evoked by NECA (5′-(*N*-ethylcarboxamido)adenosine), CPA (*N*^6^-cyclopentyladenosine) and CHA (*N*^6^-cyclohexyladenosine), three synthetic A_1_ adenosine receptor (A_1_ receptor) agonists with long half-lives, in the guinea pig atrium. We plotted *RR_%_* against *E_%_*, thereby characterizing the receptor reserve with a function. *RR_%_*/*E_%_* functions indicated considerably great A_1_ receptor reserve values for the direct negative inotropic effect [13]. In addition, the shape of these functions showed that the maximal effect, theoretically, can be achieved only at maximal receptor occupancy (that can be produced only by infinitely high agonist concentration). Thus, historical receptor reserve values published for maximal effect should actually apply to some near maximal effect. Indeed, the above-mentioned *RR_%_*/*E_%_* functions reached their maximums (80–92%) at great but not maximal *E_%_* values (90–96%) [13].

However, determining receptor reserve is challenging for rapidly metabolizing agonists, such as adenosine. Adenosine is a ubiquitous molecule of the purine metabolism, and therefore a substrate for several enzymes and carriers. Beyond (but in connection with) this, adenosine is the endogenous agonist of its own receptor family, a member of the G protein-coupled receptor superfamily [14]. Due to the general occurrence of adenosine receptors as well as the cell–surface location of their orthosteric binding site, the extracellular adenosine concentration plays a pivotal regulatory role throughout the body. Adenosine accumulates extracellularly in response to metabolic stress (predominantly ATP depletion) and cell damage. Consistent with this, adenosine receptors mediate mainly protective and regenerative effects, playing an essential role in modulating the course of several disorders and diseases [11,15,16,17]. The major adenosine receptor type of cardiomyocytes is the A_1_ receptor that initiates robust retaliatory effects (limiting energy consumption, such as negative inotropic effect) and adaptive processes (e.g., to ischemia, also reflected by the phenomenon of ischemic preconditioning) [18,19,20,21]. Accordingly, several compounds exhibiting A_1_ receptor agonist, A_1_ receptor enhancer or endogenous adenosine level-elevating properties are in the pipeline or in use for numerous indications, as antiarrhythmic, antianginal, antidiabetic and antinociceptive agents [8,9,22,23,24]. For this reason, it is important to explore the exact role of adenosine in the A_1_ adenosinergic mechanisms throughout the body that requires reliable E/c data.

All quantitative methods used to determine receptor reserve need accurate E/c data, the acquisition of which, by means of E/c curve construction, mandates the ability to reach the steady state with regard to the agonist levels at the receptors, a premise that is difficult to fulfill for an agonist with a short half-life. Moreover, reliable adenosine levels are hard to compute or measure, due to the quick adenosine turnover in most tissues including the myocardium [25,26]. Although rapid elimination of adenosine, administered to construct E/c curves for the determination, can be inhibited, the consequent accumulation of endogenous adenosine biases the result. To address this problem, we have proposed a procedure, by means of which this bias can be mathematically corrected and then the receptor reserve can be qualitatively assessed [27]. This method is closely related to the receptorial responsiveness method (RRM), which has been developed to quantify agonist concentrations in the microenvironment of the receptors (under certain circumstances) [28,29,30].

Let us illustrate our new method with the description of assessing receptor reserve for adenosine in the case of the A_1_ receptor-mediated direct negative inotropy in the guinea pig left atrium. Consistent with earlier methods [5,7,13], the essence of this procedure is the construction of adenosine E/c curves before and after a treatment with FSCPX (8-cyclopentyl-*N*^3^-[3-(4-(fluorosulfonyl)benzoyloxy)propyl]-*N*^1^-propylxanthine), an irreversible A_1_ receptor antagonist, but in the presence of NBTI (S-(2-hydroxy-5-nitrobenzyl)-6-thioinosine), an inhibitor of the equilibrative and NBTI-sensitive nucleoside transporter (ENT1). NBTI, by preventing cellular uptake and consequent intracellular elimination of adenosine, prolongs the half-life of this purine nucleoside in the guinea pig atrium [25]. In addition, it is also obligatory to generate E/c curves with a stable A_1_ receptor agonist (e.g., CPA) in the absence and presence of NBTI, and to construct an E/c curve with this stable agonist after an FSCPX treatment (but without NBTI), in order to collect data for the correction of the adenosine E/c curves subjected to NBTI (for explanation, see Section 4). The receptor reserve will be indicated by the distance of final parts of the two corrected adenosine E/c curves (constructed without and with FSCPX treatment) because the final sections of these E/c curves show the maximal effect elicited by adenosine upon unaffected and reduced A_1_ receptor populations (respectively). The closer these final parts are to each other, the greater the A_1_ receptor reserve for adenosine (and for the given tissue and the measured effect). By means of our method (using FSCPX with the recommended maximal concentration and duration time), we have found that the receptor reserve for adenosine is comparable in extent with that for NECA, CPA and CHA, three synthetic high-efficacy agonists with long half-lives, when measuring the direct negative inotropic effect mediated by the atrial A_1_ receptor [27,31].

The goal of the current study was to provide in silico validation of our new method developed to assess receptor reserve. Some E/c curves presented in our previous work [31] were simulated and then evaluated by means of this new method. The original E/c curves were generated on euthyroid guinea pig atria using adenosine, CPA, FSCPX and NBTI, with the measurement of a characteristic and robust action of the atrial A_1_ receptor, i.e., the direct negative inotropic effect [31]. For the sake of clarity, adenosine and CPA were simulated as agonist A and B, respectively, while FSCPX and NBTI were simulated as irreversible antagonist (IA) and transport inhibitor (TI), respectively.

## 2. Results

### 2.1. Features of the Simple Unbiased E/c Curves of Agonists A and B

**Figure 1:**
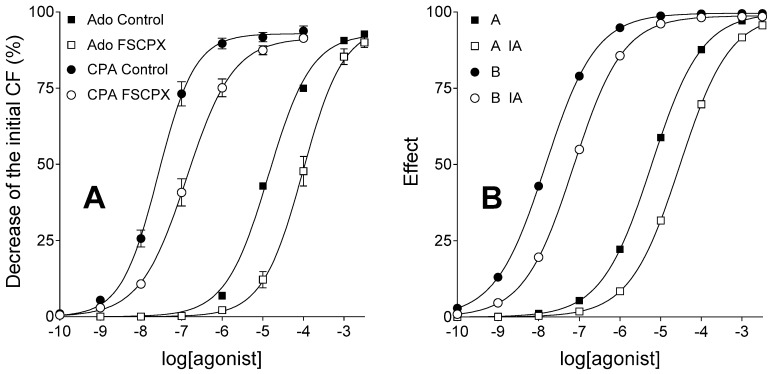
Ex vivo biological (panel **A**) and in silico simulated (panel **B**) models showing concentration-response (E/c) curves of two agonists with short (square symbols) and long (circle symbols) half-lives, acting in systems with unaffected (filled symbols) and reduced (open symbols) receptor number. The *x*-axis shows the common logarithm of the molar concentration of agonists (in the bathing medium), and the *y*-axis indicates the effect. The continuous lines denote the fitted Hill equation. On the panel **A**, symbols show mean ± SEM. Ado: adenosine (the endogenous A_1_ adenosine receptor agonist with a short half-life); CPA: *N*^6^-cyclopentyladenosine (a synthetic A_1_ adenosine receptor agonist with a long half-life); FSCPX: a prior treatment with 8-cyclopentyl-*N*^3^-[3-(4-(fluorosulfonyl)benzoyloxy)propyl]-*N*^1^-propylxanthine (an irreversible A_1_ adenosine receptor antagonist); A: agonist A (simulating adenosine); B: agonist B (simulating CPA); IA: a prior treatment with an irreversible antagonist (simulating an FSCPX pretreatment); CF: contractile force. Data of panel **A** are redrawn from [31].

E/c curves of agonist A (representing adenosine) and B (modelling CPA), simulated in a system with naïve receptor population, exhibited shape and position similar to E/c curves of adenosine and CPA (respectively), constructed using our previous data measured in FSCPX-naïve guinea pig atria. The simulated decrease in receptor number produced a moderate dextral displacement of E/c curves of agonist A and B, similar to the receptor depletion produced by a 10 µmol/L FSCPX-treatment (for 45 min followed by 75 min of washout) on the E/c curves of adenosine and CPA (Figure 1).

### 2.2. The Effect of a Treatment with TI Alone and a Co-Treatment with IA and TI on the E/c Curve of Agonist A (Biased E/c Curves of Agonist A)

**Figure 2:**
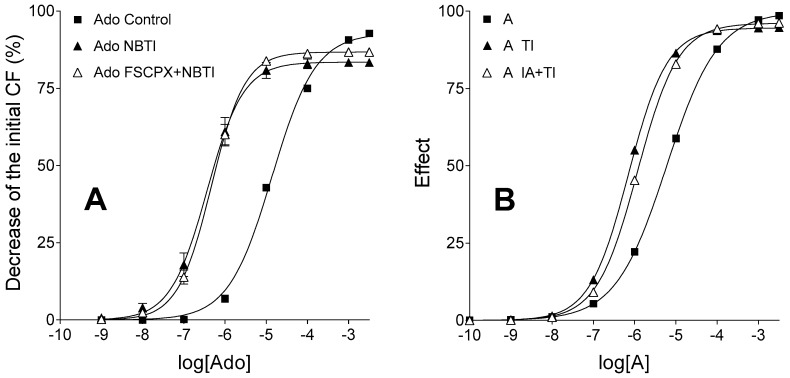
Ex vivo biological (panel **A**) and in silico simulated (panel **B**) models displaying E/c curves of an agonist with a short half-life, in the absence and presence of an agonist transport inhibitor, acting in systems with unaffected (filled symbols) and reduced (open symbols) receptor number. The real and the simulated agonist used to generate the E/c curves are both identical with the endogenous agonist of the given model that agonist is extensively transported and then eliminated. The *x*-axis denotes the common logarithm of the molar concentration of agonists (in the bathing medium), and the *y*-axis indicates the effect. The continuous lines represent the fitted Hill equation. On the panel **A**, symbols show mean ± SEM. Ado: adenosine; NBTI: a treatment with S-(2-hydroxy-5-nitrobenzyl)-6-thioinosine (an inhibitor of the nucleoside transporter type ENT1); FSCPX: a prior treatment with 8-cyclopentyl-*N*^3^-[3-(4-(fluorosulfonyl)benzoyloxy)propyl]-*N*^1^-propylxanthine (an irreversible A_1_ adenosine receptor antagonist); A: agonist A (simulating adenosine); TI: a treatment with an inhibitor of agonist A transport (simulating the presence of NBTI); IA: a prior treatment with an irreversible antagonist (simulating an FSCPX pretreatment); CF: contractile force. Data of panel **A** are redrawn from [31].

The TI treatment shifted the E/c curve of agonist A to the left and slightly diminished the maximal effect, just like a 10 µmol/L NBTI treatment did with the adenosine E/c curve. Due to the need to harmonize several factors during the simulation, this effect came out somewhat smaller in the simulated model than in the biological one. The astonishing effect of 10 µmol/L FSCPX and 10 µmol/L NBTI co-treatment on the adenosine E/c curve (i.e., upon ENT1 blockade, the irreversible A_1_ receptor antagonist appeared to increase the maximal effect of adenosine; Figure 2A), however, was not reproducible in silico under the assumption that TI elicits its action irrespectively of the IA treatment. In contrast, when we introduced an interaction between IA and TI at the level of the transmembrane transport of agonist A, the relative position of the three E/c curves could be made similar to that seen in the biological model (Figure 2B).

More specifically, we had to hypothesize that a prior IA treatment slightly blunted the effect of TI. In this case, there was a constellation of simulation parameters that enabled the IA and TI co-treated function to run first slightly under the solely TI-treated function, and then to surpass it at the highest agonist A concentrations. Importantly, both of these curves run over the simple unbiased and IA-naïve E/c curve of agonist A (representing the control curve) until the two highest concentrations, where they came below this control curve (Figure 2B).

### 2.3. The Effect of a TI Treatment on the E/c Curve of Agonist B (Biased E/c Curve of Agonist B)

**Figure 3:**
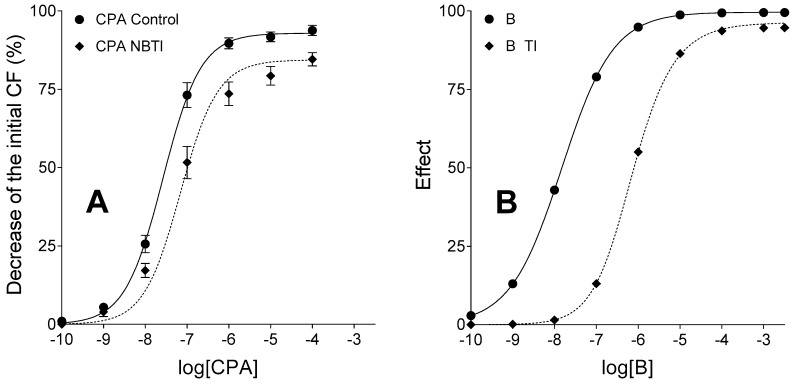
Ex vivo biological (panel **A**) and in silico simulated (panel **B**) models exhibiting E/c curves of a synthetic agonist with a long half-life, in the absence and presence of an agonist transport inhibitor, acting in a system with naïve receptor population. The transport inhibition do not affect the fate of the agonist used for the E/c curves, only the transport of the endogenous agonist (activating the same receptor as the synthetic one) was inhibited in both models. The *x*-axis indicates the common logarithm of the molar concentration of agonists (in the bathing medium), and the *y*-axis denotes the effect. The continuous lines represent the fitted Hill equation, while the dotted lines show the fitted equation of RRM (receptorial responsiveness method). On the panel **A**, symbols show the mean ± SEM. CPA: *N*^6^-cyclopentyladenosine; NBTI: a treatment with S-(2-hydroxy-5-nitrobenzyl)-6-thioinosine; B: agonist B (simulating CPA); TI: a treatment with an inhibitor of the transport of agonist A but not B (simulating the presence of NBTI); CF: contractile force. Data of panel **A** are redrawn from [31].

The TI treatment shifted the E/c curve of agonist B to the left and moderately decreased its maximal effect as compared to the simple unbiased and IA-naïve E/c curve of agonist B (representing the control curve), similarly to the CPA E/c curves generated in the presence and absence of 10 µmol/L NBTI. Although the simulated model showed somewhat different response to TI than the biological one to NBTI (probably due to the simplicity of operational model that did not allow a finer setting), the relative position of E/c curves was the same in the in silico and ex vivo models (Figure 3). Furthermore, RRM provided an estimate for the surplus agonist A concentration (in response to transport inhibition) that was almost the same as the predefined value (3.86 × 10^−7^ versus 4 × 10^−7^, respectively).

### 2.4. The Corrected E/c Curves of Agonist A (Derived from the Biased Ones)

**Figure 4:**
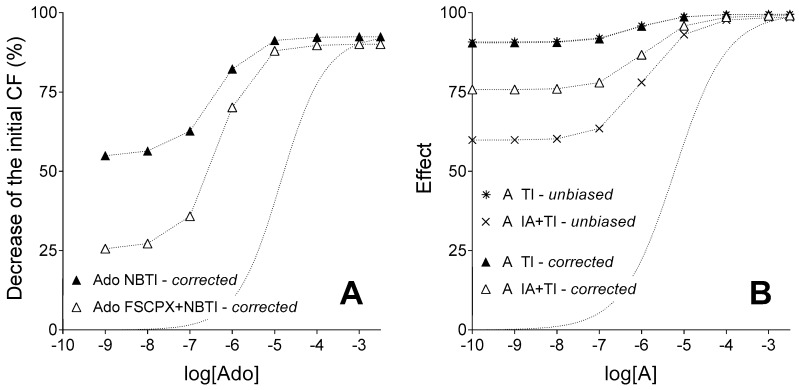
Ex vivo biological (panel **A**) and in silico simulated (panel **B**) models showing corrected E/c curves of an agonist with a short half-life, in the absence and presence of an agonist transport inhibitor, acting in systems with unaffected (filled symbols) and reduced (open symbols) receptor number (while symbols of the built-in in silico control curves labelled as “unbiased” are simply x and asterisk). The *x*-axis denotes the common logarithm of the molar concentration of agonists (in the bathing medium), and the *y*-axis indicates the effect. The dotted lines between symbols only connect them, while the dotted lines without symbols represent the Hill equation fitted to data of the control adenosine E/c curve (panel A) and the simple unbiased E/c curve of agonist A generated upon naïve receptor population (panel B). Ado: adenosine; NBTI: a treatment with S-(2-hydroxy-5-nitrobenzyl)-6-thioinosine; FSCPX: a prior treatment with 8-cyclopentyl-*N*^3^-[3-(4-(fluorosulfonyl)benzoyloxy)propyl]-*N*^1^-propylxanthine; A: agonist A (simulating adenosine); TI: a treatment with an inhibitor of agonist A transport (simulating the presence of NBTI); IA: a prior treatment with an irreversible antagonist (simulating an FSCPX pretreatment); *unbiased*: unbiased E/c curves of agonist A (control functions for the corresponding corrected E/c curves); *corrected*: E/c curves corrected with our method; CF: contractile force. Data of panel **A** are redrawn from [31].

After the correction for the biasing effect of the simulated and real agonist accumulation under agonist transport inhibition, the relative position of E/c curves exhibited the same rearrangement in the in silico and ex vivo models (Figure 4). At saturating agonist A concentrations, effect values of the IA and TI co-treated E/c curve were exceeded by effect values of the TI-treated (and IA-naïve) E/c curve, effect values of these curves being practically the same (Figure 4B). Thus, the influence of the IA treatment on the maximal effect of agonist A proved to be quite small (Figure 4B), similarly to the case in the biological model (Figure 4A). This result corroborates the finding of our previous studies, i.e., the A_1_ receptor reserve pertaining to the direct negative inotropic effect of adenosine is substantially great in the guinea pig atrium [27,31].

The corrected TI-treated (and IA-naïve) E/c curve practically overlapped its corresponding unbiased E/c curve (that served as a built-in control for the correction), as expected. In contrast, the corrected IA and TI co-treated E/c curve markedly surpassed its unbiased counterpart (the built-in control) at small and medium concentrations of agonist A. Nevertheless, at saturating agonist A concentrations, the difference between these curves disappeared (Figure 4B).

The cause of this phenomenon is the interference modelled between actions of IA and TI. To reproduce the relative position of the adenosine E/c curves, we presumed that the IA treatment slightly inhibits the otherwise complete inhibitory effect that TI has on the transport of agonist A. At the same time, according to the practice followed previously [27,31], only one *c_x_* value (the expected equieffective concentration of agonist B) was used for the quantification of the surplus concentration of agonist A produced by TI. As this *c_x_* was obtained from IA-naïve E/c curves, it informs us about the agonist accumulation under complete agonist transport blockade. Therefore, this *c_x_* could not be used to correct IA-treated E/c curves.

Since we found no prior information about any interference between FSCPX and NBTI, we applied one *c_x_* value for all calculations in our earlier studies [27,31]. Taken the results of the present study into account, this practice has proven to be theoretically objectionable. However, conclusions previously drawn from corrected E/c curves (more precisely, from the final parts of these curves indicating the maximal effect of adenosine) have remained correct. This is indicated by the observation of the present study, i.e., in the range of excessive concentrations of agonist A, a slightly bigger agonist A transport caused by IA treatment (in comparison with the condition upon complete transport inhibition) no longer significantly influences the effect evoked (Figure 4B).

## 3. Discussion

The present study has provided an in silico evidence that our qualitative method can reliably assess receptor reserve for adenosine, an endogenous agonist with rapid metabolism, in our recently published experimental setting (isolated and paced guinea pig left atrium). Furthermore, our current results indicate that the FSCPX-naïve adenosine E/c curve constructed in the presence of NBTI, which is corrected (by means of our method) for the bias caused by the accumulation of endogenous adenosine in response to NBTI, accurately reflects the effect values elicited by adenosine (of both endogenous and exogenous origin). At the same time, our findings imply that FSCPX treatment weakens the inhibitory action of NBTI on the transport (and thereby elimination) of adenosine. As a consequence, the adenosine E/c curve generated under NBTI and FSCPX co-treatment, after a correction with our method in the recently published manner [27], probably overestimates the effect of adenosine at small and medium concentrations. It is important to emphasize that this deviation affects, in a significant manner, only the starting and medium parts of this corrected E/c curve, thus it does not influence the assessment of receptor reserve for adenosine (that is based on the evaluation of final parts of the corrected E/c curves).

For the purposes of basic research as well as drug development, it is essential to explore the accurate E/c relationships for every promising receptor agonist, including compounds with short half-lives. It is also important to survey the response-inducing ability of these agents in different tissues for diverse effects mediated by the appropriate receptors. However, it is challenging to investigate quickly eliminating agents because their concentration is strongly dependent on several internal mechanisms and thereby it is sensitive to a variety of external interventions. Moreover, it is especially difficult to quantify the level of agonists with short half-lives, such as adenosine, in the most relevant tissue compartment (in terms of the E/c relationship), in the vicinity of their receptors [25,26]. These difficulties impede, among others, the quantitative determination of receptor reserve for adenosine and similar degradable agonists [13].

The present study has been designed for the simulation of adenosinergic and adenosine-handling mechanisms in the atrial myocardium, with special regard to their influence on the assessment of receptor reserve for the direct negative inotropic effect of adenosine mediated by the A_1_ receptor. Specifically, we reproduced a special set of adenosine E/c curves presented in our prior ex vivo study [31] for determining receptor reserve by means of our recently published method [27]. Then, we evaluated the simulated functions in a manner described for the ex vivo E/c curves [27,31] in order to validate our method and to ensure the best interpretation of its results.

The control, (solely) FSCPX-treated and (solely) NBTI-treated adenosine and CPA E/c curves could be easily reproduced (differences between the real and simulated curves were attributed to the generic nature of the operational model of agonism used for the simulation). However, to reproduce the position of the adenosine E/c curve subjected to FSCPX and NBTI co-treatment relative to other adenosine E/c curves (Figure 2A), it had to be assumed that the IA treatment (simulating FSCPX treatment) weakens the effect of TI (simulating NBTI), thereby it enables a bigger influx of agonist A (representing adenosine) into the cells as compared to the case of TI treatment alone. Only a small inhibitory effect afforded by IA on the TI’s action needed to be assumed for reaching the appropriate arrangement of the simulated E/c curves (Figure 2B).

The inspiring question, i.e., the exact mechanism by which FSCPX may modify the action of NBTI, has remained open during the present investigation. Herein, we have proposed a simple and plausible possibility, namely FSCPX, perhaps via a covalent modification of ENT1, allows ENT1 to work but impedes NBTI to inhibit ENT1 (so, FSCPX might act as an irreversible antagonist at the binding site of NBTI on ENT1 as well). Nevertheless, there are some other opportunities. For example, FSCPX could activate nucleoside transporters other than ENT1 (such as ENT2), an effect that would be undetectable when the dominant ENT1 works, but would become manifest upon ENT1 blockade caused by NBTI. More complex interactions involving the A_1_ receptor also cannot be excluded. To clarify the exact mechanism, further investigations are warranted.

This (newly proposed) effect of IA treatment was taken into account on the level of both exogenous and endogenous agonist A. Consequently, the corrected IA and TI co-treated E/c curve of agonist A diverged from its corresponding unbiased E/c curve (that served as the “built-in control” for the validation of our method to assess receptor reserve). It should be highlighted, however, that this divergence was the greatest at the smallest concentration of the exogenous agonist A, and it gradually decreased parallel to the increase of the exogenous agonist A concentration. On the final parts of the corrected and unbiased IA and TI co-treated E/c curves (i.e., at the highest concentrations of exogenous agonist A), only a minor difference was experienced between the corrected and unbiased effect values (Figure 4B). Therefore, conclusions drawn based on the relative position of final parts of corrected E/c curves in earlier works [27,31] can be considered to be still appropriate. This result of the present study has confirmed the existence of a great receptor reserve for the direct negative inotropic effect of adenosine mediated by the guinea pig atrial A_1_ receptor described previously [27,31]. Furthermore, it has validated our method as a reliable tool to assess receptor reserve. In addition, results of this investigation also draw attention to the obligatory precaution concerning possible unexpected effects (e.g., unanticipated interactions) of the chemicals used.

In summary, our method, presented in this and earlier studies [27,31], enables the qualitative assessment of receptor reserve for adenosine, an endogenous agonist for which no reliable receptor reserve value could be determined previously (as an example, see: [13]). Hence, this method may contribute to the accumulation of useful data regarding receptor reserve for other endogenous agonists with short half-lives (resulting from a high exposure to the function of enzymes and transporters). The potential advantage of introducing this new method into practice comes from the significance and specific nature of receptor reserve. Efficiency of different agonists may become predictable in different tissues based on the relevant receptor reserve values, a knowledge that, if utilized for rational drug development, may help diminish untoward effects. Consistently, low-efficacy A_1_ receptor agonists, proved to have a significant effect only in tissues with great A_1_ receptor reserve, have been being developed in numerous indications [8,9,22,23,24]. However, as some drugs influence the level and/or distribution of endogenous agonists, receptor reserve data related to these agonists also have therapeutical significance that indicates the raison d’être of our method to assess receptor reserve. Beyond these considerations, our method can be utilized for basic research as well, namely, to investigate the E/c relationship of endogenous agonists upon different conditions. In a prior work, based on results obtained using the correction procedure being a part of our new method (see Section 4), we proposed a new, thyroid hormone-sensitive effect of adenosine deaminase inhibition, i.e., inhibition of adenosine deaminase appeared to increase the signal amplification of the atrial A_1_ adenosinergic system, an effect that was more pronounced in hyperthyroidism (in this investigation, no irreversible A_1_ receptor antagonist was administered) [32]. This mechanism may be of practical significance in improving ischemic tolerance of the heart.

The main limitation of our method is its qualitative (at best semi-quantitative) nature. However, having a qualitative model instead of a quantitative one with inherent theoretical limitations is a trade-off, which carries considerable gains. The traditional receptor theory and methods based on it have been being criticized to oversimplify the agonist-receptor interaction. This oversimplification would hinder the calculation of valid *K_A_* values (marked with *K* in the present study, see Section 4) needed for computing exact receptor reserve values, especially in the case of the complex G protein-coupled receptors [33,34,35]. This criticism also affects the operational model [36] and Furchgott’s method [5], two procedures that are used to determine *K_A_* values for the quantification of receptor reserve (as an example, see: [13]). Moreover, quantification of affinity and efficacy parameters for an agonist-receptor interaction is always exposed to theoretical pitfalls to some extent [34,37]. However, our method is a qualitative one and is not constrained by premises of the traditional receptor theory, rather it is based on the (quite general) assumptions and requirements of the Hill equation (as receptor function model [38]) supplemented with some limits specific for RRM [28,29]. Our method exploits the phenomenon underlying the concept of receptor reserve, i.e., the extent of decrease in maximal effect in response to a treatment with an irreversible antagonist at a given concentration [27,31]. Taking all together, we feel that tailoring the concept of receptor reserve to the requisites of the post-traditional receptor theory era, including the extension of its definition beyond the receptor per se to a receptor system as a tissue-dependent functional unit (containing the receptor and the postreceptorial signaling pathways involved), the assessment of receptor reserve will have further translational and resultant clinical implications that can be utilized in rational drug development.

The use of the operational model in the present study should also be addressed. The uncertainty derived from the oversimplification of agonist-receptor interaction results in rather empirical than mechanistic *K_A_* and τ (including *K_E_*) values (see Equation (3) in see Section 4), if the operational model is fitted to E/c curve data [2,39]. In the present work, however, this model was used to generate functions shaping E/c curves, and *K_A_* (herein *K*) values of this model were not applied to calculate exact receptor reserve values.

## 4. Materials and Methods

### 4.1. Properties of the Biological Model to Be Simulated

Under physiological conditions, the myocardial adenosine metabolism is characterized by net interstitial formation and net intracellular elimination, whose processes, interconnected by several types of transmembrane nucleoside transporters, maintain a dynamic equilibrium with regard to the interstitial adenosine level [40]. In our guinea pig model, the most important adenosine carrier is ENT1. The interstitially formed adenosine rapidly enters the cells via ENT1 possessing high transport capacity [41,42] that renders the interstitial adenosine level hard to determine [25,26]. In contrast, interstitial levels of CPA, an adenosine analogue relatively resistant to adenosine-handling enzymes and carriers, proved to be easily assessable in our ex vivo atrial preparations [28].

In our guinea pig model, ENT1 blockade produced by NBTI increases the interstitial level of endogenous adenosine by minimizing the adenosine flux into the cardiomyocytes, thereby preventing the intracellular adenosine elimination [25,43]. Thus, NBTI affects the adenosine E/c curve in two opposing ways. On one hand, NBTI tends to increase the maximal response (*E*_max_) to adenosine and to decrease the adenosine concentration needed for the half-maximal response (*EC*_50_), as a result of the decreased elimination of exogenous adenosine. On the other hand, it also tends to decrease *E*_max_ and to increase *EC*_50_ because the interstitially accumulated endogenous adenosine consumes a significant portion of the response capacity of A_1_ receptors (importantly, before the construction of the E/c curve with exogenous adenosine). The net effect of these two influences is that NBTI slightly reduces *E*_max_ but markedly decreases *EC*_50_ of the adenosine E/c curve, at least in terms of the direct negative inotropy mediated by the guinea pig atrial A_1_ receptor [25,43].

On the contrary, due to the stability of CPA, the influence of NBTI on the CPA E/c curve in the same model is less complex: NBTI decreases *E*_max_ and increases *EC*_50_, a phenomenon that can be completely explained by the interstitial accumulation of endogenous adenosine in response to ENT1 inhibition that in part uses up the responsiveness of A_1_ receptors before taking the CPA E/c curve [25,43].

In summary, irrespectively of the A_1_ receptor agonist used to construct the E/c curve, the surplus interstitial endogenous adenosine produced by NBTI tends to cause a characteristic bias (i.e., depression of maximal effect and dextral displacement) on the E/c curve. However, this bias is unmasked only if this effect is the single effect of NBTI, i.e., level of the agonist used for the E/c curve is not affected by NBTI (as is the case for CPA). In general, the mechanism by which this bias develops is that the surplus endogenous agonist concentration and its effect is overlooked during the evaluation of effect values that are assigned to the agonist concentrations administered for the E/c curve. Nevertheless, the extent of this E/c curve modification provides an opportunity to quantify the neglected extra concentration of the endogenous agonist by the equieffective concentration of an agonist (optimally a stable one with a long half-life) used for the E/c curve. This concentration estimating procedure, termed RRM, is based on fitting the biased E/c curves to a specific equation (see Equation (5) below), best-fit value of which yields the equieffective concentration [25,28,43].

The main advantage of RRM is that it allows (a kind of) quantification of extra concentrations (accumulated acutely for some reason) of agonists with short half-lives that could be hardly determined in any other manner. As a limitation, RRM cannot reliably quantify the (surplus concentration of) a degradable agonist directly, rather it can provide the equieffective concentration of a stable agonist for the same receptor (or, moreover, for the same signaling pathway) [28]. An important requirement of RRM is that the surplus concentration to be estimated should be in a steady state during the procedure (a prerequisite obeyed by our experimental setting).

In this sense, three types of E/c curves are distinguished in this study: unbiased, biased and a corrected one. An E/c curve is unbiased if every agonist with its whole concentration is taken into account. In turn, an E/c curve is biased if a concentration of any agonist is neglected during the course of evaluating the effect values. (Of course, an E/c curve can be considered unbiased in all aspects if unbiased effect values are assigned to agonist concentrations evolved at the receptors, but usually this requirement does not obey for rapidly eliminating agents. This topic is not addressed in the present study.) Correction of a biased E/c curve aims to reproduce the corresponding unbiased E/c curve (at least in terms of the effect values). This procedure needs a special technique (see below).

If the overlooked extra agonist concentration is known or can be determined (e.g., with RRM), the biased E/c curve can be corrected by means of an equation (see Equation (7) below) that is a derivative of the equation of RRM (see Equation (5) below). Moreover, as a further unique feature, RRM allows the correction of a biased E/c curve even if only the equivalent concentration of another agonist (used for the E/c curve) can be determined rather than the neglected surplus agonist concentration per se [27]. This possibility is especially useful for agonists with short half-lives. Thus, the strategy of the present study was to correct the simulated biased E/c curves of a degradable agonist the same way as real biased E/c curves were corrected, and then to compare these corrected E/c curves with the corresponding unbiased E/c curves (from which the biased ones were in silico transformed).

### 4.2. Applied Mathematical Tools

1. To generate unbiased E/c curves, the operational model of agonism, a comprehensive receptor function model forming a hybrid between empirical and mechanistic models [39], was applied. Two equations of this model was used, one determining the effect of one agonist [36], and another one described for the co-action of two (different or the same) agonists [44]. In this latter equation, one agonist was present always at a single concentration, while the other one was applied in a range of concentrations. Functions provided by the equation for one agonist’s action represented simple unbiased E/c curves, while those, yielded by the equation for the co-action of two agonists, simulated unbiased E/c curves that could be easily biased by ignoring the agonist concentration used with a single value (see below). This ignored agonist concentration (defined arbitrarily for the simulation) served as a model for the surplus interstitial concentration of endogenous adenosine produced by NBTI, while increasing concentrations of the other agonist simulated the concentrations administered for the E/c curve.
2. To geometrically characterize functions via curve fitting or to calculate effect values from concentrations and best-fit values obtained by curve fitting, the Hill equation, the most widely used empirical receptor function model [38], was applied.
3. Adenosine and CPA were modelled with an agonist A and B, respectively. Therefore, a continuous extracellular production, intracellular elimination and transmembrane transport of agonist A were considered. Thus, “exogenous” agonist A concentrations (simulating the administration of agonist A for an E/c curve) were manipulated, when calculating their effect, to model the function of nucleoside transporters (see paragraph 5). Furthermore, a surplus “endogenous” agonist A concentration was designated as a response to the inhibition of nucleoside transport (for simplicity, only ENT1, i.e., an inhabitable carrier, was built into the present model). In contrast, agonist B concentrations were considered to be constant after their administration (see paragraph 5).
4. NBTI and FSCPX were represented by a so-called transport inhibitor (TI) and irreversible antagonist (IA), respectively.
5. Development of agonist concentrations was considered in two compartments. For all agonists, concentration values in the bathing medium were defined to be independent from any kind of simulated treatments. When constructing E/c curves, effect values were always plotted against the bathing medium concentrations (simulating the condition that usually these concentrations are known during ex vivo experiments). However, effect values were always computed from near-receptor concentrations defined as follows (after testing several value combinations). The bathing medium concentration was designated as a concentration at the receptors for agonist B (in all circumstances) and for agonist A under TI treatment and without IA treatment (simulating complete ENT1 inhibition). Furthermore, the bathing medium concentration divided by 400 was designated as near-receptor concentration for agonist A without IA and TI treatment (simulating the presence of intact ENT1). Finally, the bathing medium concentration divided by 3 was designated as near-receptor concentration for agonist A in the presence of TI and after an IA treatment (simulating the incomplete inhibition of ENT1). These simple operations simulated the effect of ENT1 on the concentration of adenosine, but not CPA, in the vicinity of the cell-surface A_1_ receptors, producing a parallel leftward shift of the treatment-naïve E/c curve of agonist A as compared to that of agonist B. This is in accordance with the fact that adenosine and CPA, two A_1_ receptor agonists, have practically the same *E*_max_ but very different *EC*_50_ [25].
6. Consistently, the effect of TI was simulated by omitting the division by 400, when computing the near-receptor concentration of the exogenous agonist A from the bathing medium one (see the previous paragraph), and also by taking a surplus near-receptor concentration of endogenous agonist A into account (see paragraphs 3 and 7).
7. The effect of IA treatment was simulated with a division of the total receptor concentration ([*R*_0_]) by 5 (see below). Thus, according to our previous results with FSCPX [13], it was assumed that 20% of the A_1_ receptors remained intact after IA treatment. In addition, in the case of IA and TI co-treatment, bathing medium concentrations of exogenous agonist A were divided by 3 to compute its near-receptor levels. Furthermore, a third of the value of surplus near-receptor concentration of endogenous agonist A, which was designated to simulate the treatment with TI alone, was taken into account (see paragraphs 3 and 6).
8. To simulate biased E/c curves, unbiased effect values were transformed into biased ones by means of an equation, derivative of the equation of RRM (see below) [28,29].
9. For the correction of the biased effect values, RRM was applied as described previously [27]. Briefly, the biased E/c curves of agonist B were fitted to the equation of RRM to provide information about the neglected surplus near-receptor concentration of agonist A. Using this information along with Hill parameters of the simple unbiased E/c curves of agonist B, the biased effect values were corrected by means of a rearranged form of the equation used for the biasing transformation (see paragraph 8).

### 4.3. First Step: Construction and Analysis of Simple Unbiased E/c Curves

Two systems and two agonists (A, B) were defined in terms of the operational model of agonism using the following equation (equivalent with Equation (10) in [36]):

(1) $$E=E_{m}\cdot \frac{{\left[R_{0}\right]}^{n_{op}}\cdot c^{n_{op}}}{{K_{E}}^{n_{op}}\cdot {\left(K+c\right)}^{n_{op}}+{\left[R_{0}\right]}^{n_{op}}\cdot c^{n_{op}}},$$

where *E* is the effect value; *E_m_* is the possible maximal effect; [*R*_0_] is the total receptor concentration (receptor number); *c* is the agonist concentration at the receptors; *K* is the equilibrium dissociation constant of the agonist-receptor complex (a measure for agonist affinity); *K_E_* is a measure of agonist efficacy; and *n_op_* is the operational slope factor.

After testing several value combinations, the following parameters were fixed at a constant value throughout the simulation (yielding the final in silico model): *E_m_* = 100, *K* = 3 × 10^−5^, *K_E_* = 5 × 10^−14^ and *n_op_* = 0.7.

Concentrations of agonist A and agonist B in the bathing medium ranged from 10^−10^ to 3.1623 × 10^−3^ (being logarithm of which −10 and −2.5, respectively). Near-receptor concentrations (*c* values for Equation (1)) were calculated according to the nature of simulated agonists and treatments (see the previous subsection).

The two systems were specified by [*R*_0_] equaling 10^−10^ or 2 × 10^−11^ to represent atrial samples without or with an FSCPX treatment (i.e., having naïve or reduced A_1_ receptor population), respectively.

Effect values calculated with Equation (1) were plotted versus the bathing medium concentrations of the given agonist. These E/c curves were fitted to the Hill equation (used in the following form identical with Equation (10) in [38]):

(2) $$E=\frac{E_{\max}}{1+10^{n\cdot (\log EC_{50}-\log c)}},$$

where *E* is the effect value; *E*_max_ is the maximal effect that can be elicited by the given agonist in the given system; *EC*_50_ is the agonist concentration in the bathing medium that leads to half-maximal effect; n is the Hill slope factor; and *c* is the agonist concentration in the bathing medium.

Use of the bathing medium concentrations for Equation (2) was in accordance with the curve fitting practice in the case of biological samples. Hill parameters of the E/c curves generated with agonist B in both systems were used for further analysis (see below).

### 4.4. Second Step: Computation of Unbiased Effect Values for a Subsequent Biasing Transformation

Effect values belonging to the surplus endogenous agonist A concentration acting together with increasing concentrations of exogenous agonist A or agonist B were computed by means of the following equation (equivalent with Equation (7) in [44]):

(3) $$E=E_{m}\cdot \frac{{\left(\tau_{test}\cdot c_{test}\cdot K_{bias}+\tau_{bias}\cdot c_{bias}\cdot K_{test}\right)}^{n_{op}}}{{\left(c_{test}\cdot K_{bias}+K_{test}\cdot K_{bias}+c_{bias}\cdot K_{test}\right)}^{n_{op}}+{\left(\tau_{test}\cdot c_{test}\cdot K_{bias}+\tau_{bias}\cdot c_{bias}\cdot K_{test}\right)}^{n_{op}}},$$

where *E* is the effect value; *E_m_* is the possible maximal effect; *c_bias_* is the surplus near-receptor concentration of endogenous agonist A that represents the extra endogenous adenosine concentration accumulated interstitially in response to NBTI; *c_test_* is one of the increasing concentrations of exogenous agonist A or agonist B at the receptors that models the concentration of the agonist administered for the E/c curve; *K_bias_* and *K_test_* are *K* values (see Equation (1)) for the agonist providing *c_bias_* and *c_test_*, respectively (in the final model: *K_bias_* = *K_test_* = 3 × 10^−5^); *τ_bias_* and *τ_test_* are equal to [*R*_0_]/*K_Ebias_* and [*R*_0_]/*K_Etest_*, respectively, where *K_Ebias_* and *K_Etest_* are *K_E_* values (see Equation (1)) for the agonist supplying *c_bias_* and *c_test_*, respectively (in the final model: *K_Ebias_* = *K_Etest_* = 5 × 10^−14^).

When *c_test_* values were simulated with agonist A, calculations were carried out with both [*R*_0_] values (10^−10^ and 2 × 10^−11^), and when *c_test_* was agonist B, only [*R*_0_] = 10^−10^ was considered.

The use of Equation (3) simulated the co-action of the surplus endogenous adenosine produced by NBTI and the agonist (adenosine or CPA) administered for the E/c curve. The action of TI alone was modelled, on one hand, by introducing an arbitrary *c_bias_* value into the simulation, and, on the other hand, by omitting the division of the bathing medium concentration of agonist A by 400 (that provided *c_test_* values). When TI was used together with IA, the influence of IA on the action of TI was taken into account through trisecting the originally defined value of *c_bias_* as well as the bathing medium concentration of agonist A (that resulted in *c_test_* values for this case). In the final model, *c_bias_* was fixed at 4 × 10^−7^, when simulating the use of TI alone, while it was computed as (4 × 10^−7^)/3, when simulating an IA treatment before the administration of TI.

The unbiased E/c curves of agonist A provided by Equation (3) served as a “built-in control”, when comparing them with the (biased and then) corrected E/c curves of agonist A (see below), in order to validate our recently published method to assess receptor reserve for degradable agonists [27]. In turn, the unbiased E/c curve of agonist B yielded by Equation (3) was used for correcting the biased E/c curves generated with agonist A (see below).

### 4.5. Third Step: Construction and Analysis of Biased E/c Curves

Effect values computed with Equation (3) were transformed (“biased”) using the following relationship (equivalent with Equation (5) in [28], and identical with Equation (5) in [29]):

(4) $$E\prime =100-\frac{100\cdot \left(100-E\right)}{100-E_{bias}},$$

where *E*′ is the biased effect value; *E* is the unbiased effect value provided by Equation (3); *E_bias_* is the effect elicited by *c_bias_* (computed with Equation (3) by setting *c_test_* at zero, with other parameters being the same as for the corresponding *E*).

Transformation of unbiased effect values into biased ones by means of Equation (4) simulated the neglect of *c_bias_* and its effect (*E_bias_*). Biased effect values yielded by Equation (4) were plotted against the bathing medium concentrations of the given agonist (providing *c_test_*).

Biased E/c curves of agonist A were fitted to the Hill equation (Equation (2)), as described above. In turn, the biased E/c curve of agonist B was fitted to the equation of RRM as follows (equivalent with Equations (2) and (8) in [27,28], respectively, and identical with Equation (8) in [29]):

(5) $$E\prime =100-\frac{100\cdot \left(100-\frac{E_{\max}}{1+10^{n\cdot \left(\log EC_{50}-\log\left(10^{\log c_{x}}+10^{\log c_{test}}\right)\right)}}\right)}{100-\frac{E_{\max}}{1+10^{n\cdot \left(\log EC_{50}-\log c_{x}\right)}}},$$

where *E*′ is the biased effect value; *c_x_* is the concentration of agonist B that is expected to be equieffective with *c_bias_*, the surplus near-receptor concentration of the endogenous agonist A (*c_x_* is the only best-fit value provided by Equation (5)); *E*_max_, log*EC*_50_ and n are parameters of Equation (2) fitted to the simple unbiased E/c curve of agonist B generated in the system having a naïve receptor population. Use of fitted data as input for RRM simulated the data collection in biological samples in order to quantify the effect of NBTI on the interstitial concentration of endogenous adenosine.

### 4.6. Fourth Step: Construction of Corrected E/c Curves from the Biased E/c Curves of Agonist A

Effect values belonging to *c_x_* were computed by means of the Hill equation (in the following form identical with Equation (3) in [27]):

(6) $$E_{x}=E_{\max}\cdot \frac{{c_{x}}^{n}}{{c_{x}}^{n}+E{C_{50}}^{n}},$$

where *E_x_* is the effect value elicited by *c_x_*; *c_x_* is the agonist B concentration expected to be equieffective with *c_bias_* (provided by Equation (5)); *E*_max_, *EC*_50_ and n are parameters of Equation (2) fitted to the simple unbiased E/c curves of agonist B in both systems (with normal and reduced receptor number, see below). These latter parameters describe the E/c relationship between agonist B and the two systems (in a way that is also achievable for biological samples).

*E_x_*, computed with Equation (6), simulated the effect evoked solely by the extra concentration of endogenous agonist accumulated by NBTI in the biological model. When *E_x_* was computed for the system with a naïve receptor population, Hill parameters of the simple unbiased E/c curve of agonist B generated in the system with normal receptor number were substituted into Equation (6). In turn, when *E_x_* was calculated for the system with reduced receptor number, Hill parameters of the simple unbiased E/c curve of agonist B constructed in the system with reduced receptor population were used for Equation (6).

Biased effect values computed with Equation (4) (where *c_test_* was agonist A) were corrected by means of the rearranged form of Equation (4) as follows (equivalent with Equation (4) in [27]):

(7) $$E_{corr}=100-\frac{\left(100-E\prime )\cdot (100-E_{x}\right)}{100},$$

where *E_corr_* is the corrected effect value that is expected to equal the corresponding unbiased effect value; *E*′ is the corresponding biased effect value; and *E_x_* is the effect value solely elicited by *c_x_* (according to Equation (6)).

The corrected effect values yielded by Equation (7) were plotted against the bathing medium concentrations of agonist A.

### 4.7. Computer Simulation and Data Analysis

According to the recommendation, agonist concentrations, *EC*_50_ and *c_x_* in the equations used for curve fitting (i.e., Equations (2) and (5)) were expressed as common logarithms [45]. Effect values of Equations (1), (3), (4), (6) and (7), together with *E_bias_* in Equation (4), were computed with Microsoft Excel 2013 (Microsoft Co., Redmond, WA, USA). For curve plotting and fitting, GraphPad Prism 7.03 for Windows (GraphPad Software Inc., La Jolla, CA, USA) was used.

## 5. Conclusions

The present study has provided an in silico validation for determining receptor reserve for adenosine, an endogenous agonist with rapid metabolism, by means of our qualitative method in a recently published experimental setting (isolated and paced guinea pig left atrium). Furthermore, this study pointed to the importance of in silico validation to disclose or exclude unforeseen interactions between treatment schedules (a problem that can occur in every study), and, in general, to shine a light on previous results. In addition, our results suggest that FSCPX and NBTI may interact in a way in which full inhibition of ENT1 elicited by NBTI is blunted by FSCPX. To the best of our knowledge, this observation has no antecedents in the literature.
